# Infection with *Mycobacterium lepromatosis*

**DOI:** 10.4269/ajtmh.16-0473

**Published:** 2016-09-07

**Authors:** David M. Scollard

**Affiliations:** ^1^ National Hansen's Disease Programs, Baton Rouge, Louisiana

Microbiologically speaking, the ancient scourge of leprosy has a new face. Considered a curse for millennia, the causative agent, *Mycobacterium leprae*, was first described by Armauer Hansen in 1874. This inaugurated the field of medical microbiology, preceding the discovery of *Mycobacterium tuberculosis*. But *M. leprae* is not cultivable, a fact that has greatly hindered many of the basic studies that enabled rapid progress in understanding other infectious diseases. Sequencing of the full *M. leprae* genome^1^ ushered in a new era in the microbiology of this disease, and it was not long before molecular studies identified a genetically similar organism associated with leprosy, dubbed “*Mycobacterium lepromatosis*.”^2^

The report in this issue by Sotiriou and others (two cases of leprosy in siblings caused by *M. lepromatosis* and review of the literature)^3^, of *M. lepromatosis* infection in two siblings, provides the best clinical descriptions to date of the syndrome caused by this organism, which appears to be clinically equivalent in all respects to the classical disease, leprosy. Both of these patients had the lepromatous form of the disease with a high bacterial load, and had successful but slow resolution of the infection after initiation of treatment with standard multidrug treatment regimens for leprosy. Both siblings experienced leprosy reactions, which are host-determined immunological phenomena well known in leprosy, and these also responded to standard immunosuppressive treatment. The occurrence of *M. lepromatosis* in siblings may indicate a common source of infection, but may also be related to shared determinants of genetic susceptibility, since host susceptibility among siblings is well described in leprosy.^4^

Microbiologically, *M. lepromatosis* is very similar to *M. leprae*: both are acid-fast, non-cultivable, and have the ability to infect peripheral nerves. The similarity of their genomes prompted initial consideration that this might represent a strain of *M. leprae* versus a new species.^5^ The *M. leprae* genome itself is very highly conserved, and since the full sequence of *M. lepromatosis* shows an overall difference of 9%,^6^ it is now recognized as a new species. Tentative molecular identification of *M. lepromatosis* should be confirmed by sequencing, to avoid the possibility of false-positive results with other techniques. The similarities between these two organisms, microbiologically and clinically, have prompted Singh and others^6^ to propose that they represent an “*M. leprae* complex,” analogous to the *Mycobacterium* species that constitute the tuberculosis complex.

Contrary to some initial reports, *M. lepromatosis* does not appear to be more ominous than its close relative, *M. leprae*. Based on the limited experience of the National Hansen's Disease Programs (NHDP) with the treatment and follow-up of a small number of patients with this infection in the United States and Canada, it appears that infection with *M. lepromatosis* manifests clinically and histologically with a wide spectrum of lesions that is well described in leprosy. Similarly, the bacterial load in different patients ranges from low to high, again a typical feature of leprosy. Both infections respond to the same treatment, and have the same prognosis, that is, some patients develop reactions and others do not.

As described in the excellent report by Sotiriou and others, *M. lepromatosis* has been reported in a patient in Canada, as well as in archival biopsy specimens from patients in several provinces in Mexico. The NHDP is also aware of cases arising in patients from countries in Central America. Remarkably, this organism has also been reported in red squirrels in Scotland.^7^ The significance of such an animal infection is not yet known, but it does suggest the possibility that in addition to the well documented *M. leprae* zoonosis in armadillos,^8^,^9^ there may be other natural reservoirs of *M. leprae*-complex organisms. The existence of zoonotic infection with *M. leprae*, and possibly with other members of the *M. leprae* complex, appears to constitute a major challenge to the World Health Organization's paradigm for leprosy elimination, which is based entirely on interruption of human–human transmission. The current paradigm does not address zoonotic transmission of any kind.

Clinically, the evidence thus far indicates that differentiation of *M. lepromatosis* from *M. leprae* in individual patients does not appear to be necessary for diagnosis or prescription of optimal medical treatment, since these organisms respond well to the same antimycobacterial regimens. However, we still know relatively little about *M. lepromatosis*, and additional work is needed to determine if it can be propagated in the mouse footpad or other animal models, so that it can be studied more extensively. Nevertheless, the identification of *M. lepromatosis* is likely to be of value in epidemiological studies, as a key in the newly developing array of molecular tools for the identification of the *M. leprae* complex, including several recently defined *M. leprae* genotypes.^8^,^9^ Together, these tools have great potential value in enabling a better understanding of the epidemiology of leprosy, addressing issues of transmission including the possibility of environmental sources and potential animal hosts.
